# Biochemical analysis of pistol self-cleaving ribozymes

**DOI:** 10.1261/rna.052514.115

**Published:** 2015-11

**Authors:** Kimberly A. Harris, Christina E. Lünse, Sanshu Li, Kenneth I. Brewer, Ronald R. Breaker

**Affiliations:** 1 Howard Hughes Medical Institute, Yale University, New Haven, Connecticut 06520-8103, USA; 2 Department of Molecular, Cellular and Developmental Biology, Yale University, New Haven, Connecticut 06520-8103, USA; 3 Department of Molecular Biophysics and Biochemistry, Yale University, New Haven, Connecticut 06520-8103, USA

**Keywords:** comparative sequence analysis, phosphoester transfer, phosphorothioate, RNA processing, RNA cleavage

## Abstract

Pistol RNAs are members of a distinct class of self-cleaving ribozymes that was recently discovered by using a bioinformatics search strategy. Several hundred pistol ribozymes share a consensus sequence including 10 highly conserved nucleotides and many other modestly conserved nucleotides associated with specific secondary structure features, including three base-paired stems and a pseudoknot. A representative pistol ribozyme from the bacterium *Lysinibacillus sphaericus* was found to promote RNA strand scission with a rate constant of ∼10 min^−1^ under physiological Mg^2+^ and pH conditions. The reaction proceeds via the nucleophilic attack of a 2′-oxygen atom on the adjacent phosphorus center, and thus adheres to the same general catalytic mechanism of internal phosphoester transfer as found with all other classes of natural self-cleaving ribozymes discovered to date. Analyses of the kinetic characteristics and the metal ion requirements of the cleavage reaction reveal that members of this ribozyme class likely use several catalytic strategies to promote the rapid cleavage of RNA.

## INTRODUCTION

Self-cleaving ribozymes are widespread across all domains of life and their architectural diversity is unmatched by any other type of natural catalytic RNA. These ribozymes have the ability to cleave their ribose-phosphate backbone at specific sites via internal phosphoester transfer with rate constants that typically exceed 10 million fold over that of spontaneous RNA degradation. The nine known self-cleaving ribozyme classes are able to accomplish this function by using a variety of structures that form unique catalytic cores (Doherty and Doudna 2000; Ferré-D'Amaré and Scott 2010; Eiler et al. 2014; Liu et al. 2014; Ren et al. 2014).

**FIGURE 1.**
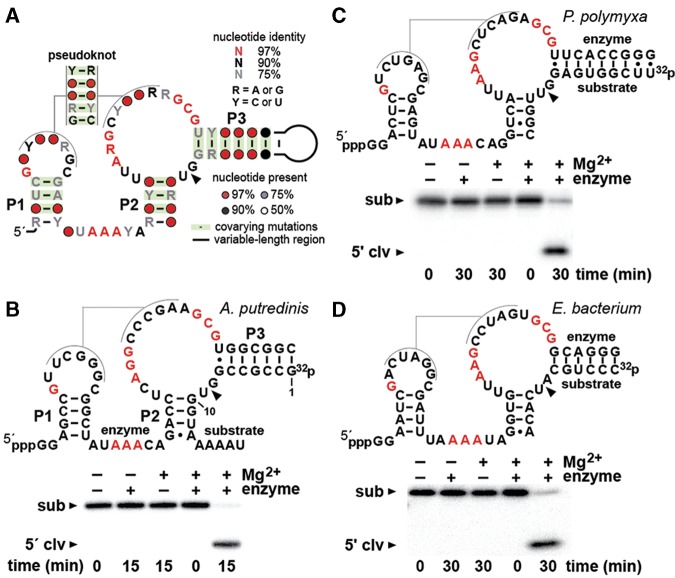
Sequence, structure, and activity of pistol ribozymes. (*A*) Consensus sequence and secondary structure model for pistol ribozymes based on the alignment of 500 unique representatives found in DNA sequence databases comprised of complete bacterial genomes, microbial metagenomic DNA, and bacteriophage DNA. The arrowhead designates the ribozyme-mediated cleavage site. (*B*) (*Top*) Sequence and predicted secondary structure of the bimolecular pistol ribozyme construct derived from *A. putredinis.* Nucleotides in red correspond to the highly conserved positions from the consensus model. (*Bottom*) Self-cleavage activity of the *A. putredinis* bimolecular ribozyme construct. The ^32^P-labeled substrate RNA (∼1 nM) was incubated for the times indicated at 23°C in the presence (+) or absence (−) of 20 mM MgCl_2_ and unlabeled enzyme RNA (∼100 nM) (see Materials and Methods for details). The cleavage products were separated by denaturing 20% PAGE. The full-length substrate (sub) and 5′-cleavage product (5′ clv) are denoted. (*C*) Sequence, secondary structure, and activity of a bimolecular pistol ribozyme construct from *P. polymyxa*. Annotations are as described in *A* and *B*. (*D*) Sequence, secondary structure, and activity of a bimolecular pistol ribozyme construct from *E. bacterium*. Annotations are as described in *A* and *B*.

Recently, we described a search strategy utilizing a bioinformatics pipeline that resulted in the discovery of three previously undiscovered classes of self-cleaving ribozymes (Weinberg et al. 2015). The pistol ribozyme class (Fig. 1A) and two other distinct ribozyme classes, called twister sister and hatchet, were uncovered using a computational approach that was specifically designed to identify novel self-cleaving ribozymes. This approach exploited the fact that the genomic DNA templates for the transcription of some known self-cleaving ribozyme classes, such as hammerhead (Prody et al. 1986) and twister (Roth et al. 2014), are commonly associated with specific genetic elements such as certain phage genes and genes for other self-cleaving ribozymes (Weinberg et al. 2015).

Preliminary analysis of a representative pistol RNA demonstrated that it performs site-specific self-cleavage in the presence of Mg^2+^, and can accelerate RNA strand scission many orders of magnitude above the uncatalyzed rate constant for RNA cleavage (Weinberg et al. 2015). In the current study, we present a more detailed biochemical analysis of members of the pistol ribozyme class. Bimolecular constructs of pistol RNAs from four different organisms were examined. Kinetic and structural analyses confirm the importance of conserved sequence and structural features, which supports the original consensus model and the conclusion that pistol RNAs represent a distinct ribozyme class. Our results also indicate that pistol ribozymes use multiple catalytic strategies to generate the large rate enhancements observed.

## RESULTS AND DISCUSSION

### Consensus sequence and structure model of pistol ribozymes

Pistol RNAs frequently appear near bacteriophage-related genes, and are found prevalently in the firmicutes phylum and in DNA sequences collected from environmental samples. The propensity for pistol RNA association with bacteriophages is not surprising. The first members of this RNA class were discovered by focusing our computational searches on intergenic regions near a collection of ribozyme-associated genes that include several phage-associated proteins (Weinberg et al. 2015).

The original consensus sequence and secondary structure model for the pistol ribozyme class was created based on the alignment of 449 unique examples (Weinberg et al. 2015). Using the latest DNA sequence databases, we identified an additional 51 unique examples, including several additional representatives encoded by bacteriophage genomes. These additional representatives were added to the pistol RNA alignment, and an updated consensus sequence and secondary structure model of pistol ribozymes was generated (Fig. 1A). This consensus model retains all of the key features of the original model. Specifically, the RNAs have the ability to form three base-paired stems (P1, P2, and P3) and a pseudoknot formed between the loop of P1 and the junction linking the left shoulders of P2 and P3. Initial evidence for the existence of these substructures was derived from the observation of extensive nucleotide covariation among the representatives. This covariation trend that commonly retains base-pairing is also evident in the expanded list of representatives.

Ribozyme-mediated cleavage occurs between a modestly conserved GU dinucleotide that also serves as the junction linking the right shoulders of P3 and P2 (Weinberg et al. 2015). Although the identity of these two nucleotides varies somewhat among pistol RNAs, the length of this junction remains strictly conserved. Moreover, the additional representatives found by our more exhaustive database searches still retain the 10 highly conserved nucleotides, which are likely critical for promoting high-speed catalysis. In our earlier study, we demonstrated that the mutation of two of these highly conserved nucleotides abolishes catalytic activity (Weinberg et al. 2015), thereby demonstrating their integral role in ribozyme structure formation or as direct participants in the RNA cleavage mechanism.

### Pistol ribozymes cleave via internal phosphoester transfer

Previously, we demonstrated that a bimolecular construct of a pistol RNA representative from *Alistipes putredinis* cleaves in vitro and we established its cleavage site (Weinberg et al. 2015). To expand on these initial findings, a similar bimolecular pistol RNA construct from *A. putredinis* was created by dividing the motif into two strands. Separate enzyme and substrate RNA strands were designed to form a bimolecular construct wherein the loop of P3 was deleted (Fig. 1B, top). When the ^32^P-labeled substrate strand containing the cleavage site is combined with the enzyme strand containing the vast majority of the conserved nucleotides, the substrate RNA is cleaved to near completion only in the presence of Mg^2+^ (Fig. 1B, bottom). Two other bimolecular pistol RNAs from *Paenibacillus polymyxa* (Fig. 1C) and *Erysipelotrichaceae bacterium* (Fig. 1D) were tested to verify the activity of additional members of this class. Likewise, both constructs promote substrate cleavage only in the presence of Mg^2+^.

**FIGURE 2.**
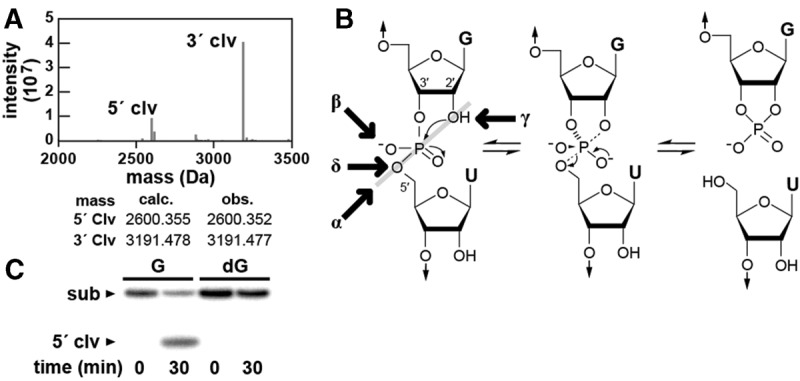
Pistol ribozyme cleavage products and the importance of the 2′-oxygen nucleophile at the cleavage site. (*A*) Mass spectrometry of the cleavage products from the *A. putredinis* bimolecular pistol ribozyme. Peaks corresponding to the expected 5′-cleavage (5′ clv) and 3′-cleavage (3′ clv) products are annotated, and the observed (obs.) and calculated (calc.) masses for these peaks are presented. (*B*) Catalytic strategies that can be used to promote the internal phosphoester transfer mechanism for RNA cleavage. Catalytic strategies include: α, the arrangement of the 2′-oxygen, phosphorus and 5′-oxygen atoms for in-line nucleophilic attack; β, neutralization of the negative charge on the nonbridging phosphate oxygen; γ, deprotonation of the 2′-hydroxyl group; δ, neutralization of the developing charge on the 5′-oxygen atom. (*C*) Ribozyme activity with an all-RNA substrate (G) (see also Fig. 1B) and a substrate analog wherein a 2′-deoxyguanosine is substituted for the guanosine ribonucleotide at position 8 of the substrate RNA (dG). Bimolecular reactions were incubated and analyzed as described in the legend of Figure 1B.

The cleavage reaction of the *A. putredinis* pistol ribozyme yields a 5′ cleavage product with a terminal 2′,3′-cyclic phosphate and a 3′ cleavage product with a 5′ hydroxyl group as determined by mass spectrometry (Fig. 2A). These cleavage products suggest that the reaction likely occurs through an internal phosphoester transfer mechanism wherein the 2′-hydroxyl group of G8 in the substrate RNA attacks the adjacent phosphorus resulting in the departure of the 5′ oxygen of U9 (Fig. 2B). This is a common mechanism exploited by all known natural self-cleaving ribozymes (Emilsson et al. 2003; Ferré-D'Amaré and Scott 2010; Roth et al. 2014; Weinberg et al. 2015). As expected, a substrate RNA that lacks the 2′ oxygen atom at G8 (the putative nucleophile) is unable to cleave under reaction conditions that permit cleavage of the all-RNA substrate (Fig. 2C).

**FIGURE 3.**
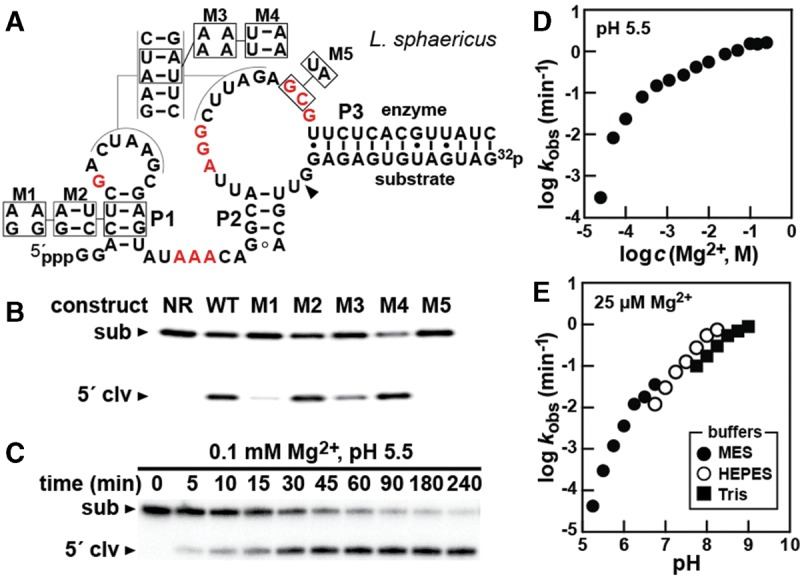
Kinetic characteristics of a pistol ribozyme. (*A*) Sequence and predicted secondary structure of the bimolecular construct derived from *L. sphaericus*. Altered nucleotides in mutant (M1, M3, M5) and compensatory mutant (M2, M4) constructs are denoted by boxes. The arrowhead designates the ribozyme-mediated cleavage site. Red nucleotides are highly conserved in the consensus model. (*B*) Self-cleavage activity of WT and mutant constructs with ^32^P-labeled substrate RNA. (NR) No reaction for the WT bimolecular construct. All other reactions were incubated under standard reaction conditions for 10 sec. (*C*) A representative time course for the bimolecular ribozyme construct under the conditions indicated as used for determining *k*_obs_ values. (*D*) Effect of Mg^2+^ concentration on the rate of pistol ribozyme cleavage. (*E*) Effect of pH on the rate of pistol ribozyme cleavage.

To biochemically validate the proposed secondary structure depicted in the consensus model, mutations in the P1 stem and the pseudoknot of a bimolecular construct from *Lysinibacillus sphaericus* were prepared (Fig. 3A). Mutations M1 and M3 that disrupt predicted base-paired elements P1 and the pseudoknot cause a substantial loss of ribozyme activity (Fig. 3B). In contrast, mutations M2 and M4 that carry nucleotides that restore base-pairing (but that are distinct from wild type) also restore ribozyme activity. The M5 construct includes mutations at two highly conserved nucleotides, and the ribozyme activity of this construct is completely abolished. These results are consistent with the proposed secondary structure and suggest that both the short P1 stem and the pseudoknot, which in the primary structure are a considerable distance from the cleavage site, are very important for ribozyme activity.

### Kinetic characteristics of a pistol ribozyme

The influence of Mg^2+^ concentration and pH on the speed of pistol ribozyme cleavage was assessed by again using the bimolecular ribozyme construct from *L. sphaericus* (Fig 3A). For this analysis, rate constants were determined by monitoring the amount of substrate RNA cleaved over time for each reaction condition examined. Ribozyme reactions were initiated by the addition of MgCl_2_, aliquots were removed to halt reactions at certain time points, and the amount of substrate cleavage was determined after product separation by polyacrylamide gel electrophoresis (PAGE) (e.g., Fig. 3C).

Initial experiments using near physiological concentrations of Mg^2+^ and pH yielded ribozyme speeds that were far too fast to accurately establish rate constants via manual removal of reaction aliquots. Therefore, we separately employed either suboptimal Mg^2+^ concentrations or suboptimal pH conditions to slow the reactions. Following separation of the reaction products by PAGE, band intensities of the radiolabeled substrate and product were quantified and the fraction of substrate cleaved was plotted versus time. The absolute value of the initial slope of the data points represents the observed rate constant (*k*_obs_).

The log–log plot of the *k*_obs_ values measured at pH 5.5 versus varying MgCl_2_ concentrations reveals that the rate constant for RNA cleavage increases with increasing amounts Mg^2+^, but begins to plateau at concentrations above ∼50 mM (Fig. 3D). The initial slope of this curve is >1, suggesting that at least two divalent metal ions are required to promote proper folding and function of the ribozyme. However, the more modest increase in RNA cleavage activity observed with concentrations of divalent metal ion >1 mM suggests that physiological concentrations of Mg^2+^ are sufficient for the ribozyme to attain near maximal activity.

The effect of pH on the rate constant is depicted in a log–log plot of *k*_obs_ values measured at 25 μM MgCl_2_. The *k*_obs_ values of the ribozyme increase as the pH of the reaction is increased. Notably, the plotted data have a slope of ∼2 between pH 5.0 and 6.25, whereas at pH values higher than 6.25, the slope is ∼1. The simplest explanation for this pattern is that at least two deprotonation events are necessary for ribozyme activity. If this hypothesis is true, then the p*K*_a_ of one of these functional groups is near 6.5, whereas the other p*K*_a_ is above 8. However, many other more complex scenarios are possible that also could explain the pH-dependent characteristics of this ribozyme.

If the Mg^2+^ and pH effects are independent of each other, which is true for hammerhead ribozymes (Canny et al. 2004), then an estimate for the rate constant under physiolocial conditions can be accurately made. For this pistol construct, the *k*_obs_ is predicted to be >10 min^−1^ under simulated physiological conditions (1 mM MgCl_2_, pH 7.5). Under optimal reaction conditions, (Mg^2+^ concentration above 50 mM and pH between 7.5 and 9.0), the rate constant is projected to be >100 min^−1^.

### Effects of di- and monovalent metal ions on pistol ribozyme cleavage

**FIGURE 4.**
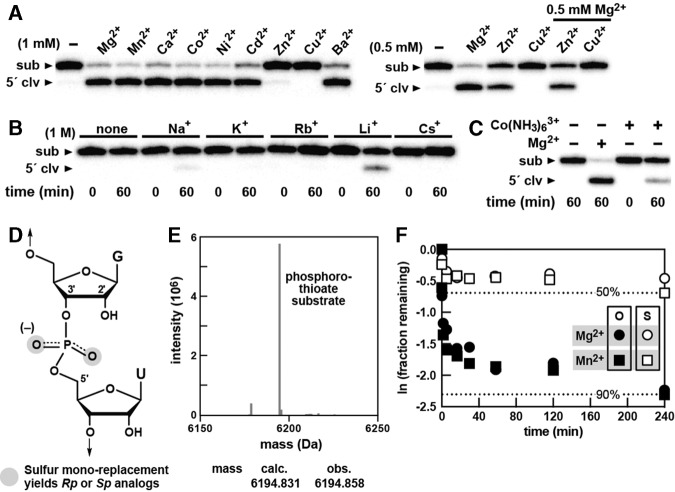
Metal ion dependency of a pistol ribozyme. (*A*, *left*) Pistol ribozyme cleavage assays with the *L. sphaericus* bimolecular construct (Fig. 3A) incubated for 30 min in absence (−) or presence (+) of various divalent metal ions at concentrations of 1 mM. (*Right*) Incubation of the bimolecular pistol construct for 30 min with 0.5 mM divalent metal ions alone or in combination with equal amounts of MgCl_2_. (*B*) Incubation of the same bimolecular construct in the absence (none) or presence of different monovalent cations at 1 M for 1 h. To chelate contaminating divalent metal ions, 30 mM EDTA was added to these reactions. (*C*) Reactions of the bimolecular complex in the absence (−) or presence (+) of 5 mM cobalt hexammine chloride [Co(NH_3_)_6_Cl_3_] or MgCl_2_ for 1 h in the presence of 5 mM EDTA. (*D*) Chemical structure of a phosphorothioate RNA linkage. The gray circles highlight the fact that the phosphorothioate substrate analogs exist as a mixture of two isomers, *R*_P_ and *S*_P_. (*E*) Mass spectrum depicting the *A. putredinis* phosphorothioate substrate RNA for the bimolecular pistol ribozyme construct. The correlation between the calculated (calc.) and observed (obs.) masses of the modified RNA is consistent with the presence of a sulfur atom. (*F*) Self-cleavage analysis of the *L. sphaericus* pistol ribozyme with either a phosphate (O) or phosphorothioate (S) linkage at the cleavage site depicted by a plot of the natural logarithm of the fraction of substrate remaining versus time. Cleavage assays were performed in standard reactions conditions with either 1 mM MgCl_2_ or 0.1 mM MnCl_2_. Dashed lines designate *y*-axis values that represent 50% or 90% cleavage of the substrate.

Cationic metals play critical roles in promoting RNA folding and enhancing structure stability. For ribozymes, metal ions typically can have two major functions: They support folding of the RNA into an active structure and they are sometimes located at the active site where they are directly involved in the catalytic mechanism (Fedor 2002). To examine the metal ion specificity of pistol ribozymes, individual monovalent and divalent metal ions were added to reaction mixtures of the bimolecular *L. sphaericus* pistol ribozyme construct, and single time-point cleavage assays were conducted (Fig. 4).

Most divalent metal ions examined support cleavage activity of the *L. sphaericus* pistol ribozyme. At a concentration of 1 mM, ribozyme cleavage can be observed with Mg^2+^, Mn^2+^, Ca^2+^, Co^2+^, Ni^2+^, Cd^2+^, and Ba^2+^ (Fig. 4A, left). These results suggest that divalent metal binding pockets formed by the RNA are relatively nonspecific. In contrast, Zn^2+^ and Cu^2+^ do not independently support robust ribozyme activity when tested at 1 mM. However, Zn^2+^ was found to support ribozyme cleavage at 0.5 mM (Fig. 4A, right), suggesting that higher concentrations of this divalent cation disrupt the active structure of the ribozyme. We observed that 0.5 mM Cu^2+^ inhibits ribozyme cleavage even in the presence of an equal concentration of Mg^2+^. This is not surprising since copper ions are known to strongly bind to biopolymers and disrupt the functions of structured RNAs at concentrations similar to those used herein (Rifkind et al. 1976; Carmi and Breaker 2001; Zivarts et al. 2005).

In the absence of Mg^2+^, ribozyme cleavage is only modestly supported by monovalent sodium and lithium ions (Fig. 4B, 5% and 24% processing, respectively, in 60 min), which have small ionic radii. In contrast, monovalent ions with larger ionic radii including Cs^+^, Rb^+^, and K^+^, do not promote ribozyme activity. The fact that a pistol ribozyme cleaves RNA in the presence of monovalent ions alone, even with only a modest rate enhancement, suggests that direct divalent metal ion participation is not an absolute requirement. This result indicates that at least some of the rate enhancement generated by members of this ribozyme class does not require Mg^2+^, which supports the hypothesis that divalent ions might serve a purely structural role.

Although, this pistol ribozyme does not appear to have a stringent requirement for Mg^2+^, it is possible that one or more divalent metal ion binding sites require inner-sphere contact with Mg^2+^ for optimal activity. Therefore, we assessed the effects of cobalt hexammine on ribozyme cleavage. Cobalt hexammine is structurally analogous to fully hydrated Mg^2+^, but the amine ligands of cobalt cannot be easily displaced by other ligands (Cowan 1993). Therefore, cobalt hexammine cannot substitute for Mg^2+^ ions in binding pockets where inner-sphere coordination is critical. Our assays revealed that the bimolecular *L. sphaericus* pistol ribozyme exhibits modest RNA cleavage activity in the presence of 5 mM cobalt hexamine (Fig. 4C), suggesting that ribozyme function does not require inner-sphere contacts with a divalent metal ion.

The possible roles of divalent metal ions were further explored by assessing whether inner-sphere coordination of Mg^2+^ by a nonbridging phosphate oxygen at the cleavage site might be utilized as a catalytic strategy to enhance the rate constant for RNA cleavage. A substrate RNA was synthesized with a phosphorothioate modification at the cleavage site, in which one of the two nonbridging phosphate oxygen atoms is replaced by sulfur (Fig. 4D,E). Cleavage reactions were performed with the bimolecular WT construct wherein the phosphorothioate RNA substrates were incubated with enzyme strands in the presence of either MgCl_2_ or MnCl_2_ (Fig. 4F). Mg^2+^ has a much higher affinity for oxygen than sulfur, while Mn^2+^ has equal affinity for both (Jaffe and Cohn 1979). Therefore, ribozymes with a nonbridging phosphate oxygen at the cleavage site should cleave with a similar rate constant and to a similar extent with either metal ion. However, ribozymes with a nonbridging sulfur atom should only be able to cleave efficiently in the presence of Mn^2+^ if inner-sphere metal ion coordination is critical at this atom. Because the phosphorothioate substrate preparation should be composed of a near equal fraction of *R*_P_ and *S*_P_ isomers, only about half of the substrate RNAs are expected to be cleaved with Mg^2+^ if inner-sphere coordination is important. Moreover, if direct metal ion coordination is required, Mn^2+^ should be able to facilitate RNA phosphorothioate cleavage beyond 50% cleavage yield.

As expected, the reactions performed with unmodified substrate and either MgCl_2_ or MnCl_2_ cleaved to ∼90% after exhaustive incubation (Fig. 4F). Surprisingly, the phosphorothioate substrate was only cleaved to ∼50% in the presence of either MgCl_2_ or MnCl_2_. For both substrates, the initial *k*_obs_ values are nearly equal when incubated with either MgCl_2_ or MnCl_2_ (2 min^−1^ and 3 min^−1^, respectively). These findings suggest that pistol ribozymes exploit a critical contact between the ribozyme active site and one of the two nonbridging phosphate oxygen atoms. However, a sulfur atom at this position is sufficient to disrupt this contact and hinder ribozyme activity in a manner that cannot be restored by the presence of Mn^2+^. Therefore, assuming that the sulfur-containing RNA construct is properly folded, a functional group from RNA (rather than an active site divalent metal ion) appears to be most likely to form this contact.

### Possible catalytic strategies used by pistol ribozymes

Four major catalytic strategies can be used by enzymes to promote RNA cleavage via internal phosphoester transfer (Breaker et al. 2003; Emilsson et al. 2003), although ribozymes can employ a diversity of means to execute these strategies (Cochrane and Strobel 2008; Ferré-D'Amaré and Scott 2010; Lilley 2011). The four strategies include the arrangement of the 2′-oxygen nucleophile, phosphorus electrophile, and 5′-oxygen leaving group in an in-line geometry (α), neutralization of the negative charge on a nonbridging phosphate oxygen (β), deprotonation of the 2′-hydroxyl group (γ), and neutralization of the developing negative charge on the 5′-oxygen leaving group (δ) (Fig. 2B). Either one or a combination of these strategies can be used by enzymes to help stabilize the transition state of the reaction and enhance reaction rate constants far beyond that of the uncatalyzed spontaneous reaction.

Previously, it has been suggested that a collection of engineered RNA-cleaving ribozymes and deoxyribozymes use only two of these strategies, specifically α and γ, to approach a theoretical maximum rate constant of ∼2 min^−1^ (Breaker et al. 2003). To exceed this αγ speed limit, enzymes must make use of β or δ catalysis in combination with additional catalytic strategies (Emilsson et al. 2003). Our analysis of the kinetic characteristics of the reaction catalyzed by the *L. sphaericus* RNA construct suggests that pistol ribozymes are able to reach speeds of >100 min^−1^ under optimal conditions. Given that the rate constant exceeds the speed limit predicted for enzymes that exclusively employ α and γ catalytic strategies, it seems likely that pistol ribozymes use β and/or δ catalysis to accelerate RNA cleavage.

Moreover, given that a pistol ribozyme construct also sharply discriminates against one of the two phosphorothioate substrate isomers (Fig. 4F), we conclude that pistol ribozymes most likely exploit β catalysis, in addition to other catalytic strategies, to achieve maximal activity. Also, it appears that the activity of the ribozyme with the phosphorothioate substrate cannot be recovered in the presence of the more thiophilic Mn^2+^. This finding suggests that pistol ribozymes might not employ a metal ion to neutralize the negative charge on the nonbridging phosphate oxygen. Due to the inherent challenges in deciphering the importance of various catalytic strategies on enzyme functions, it will be very useful to collect and evaluate structural data to further elucidate the details of the active site of pistol ribozymes.

## CONCLUSIONS

Bioinformatic and biochemical analyses demonstrate that pistol RNAs are members of a novel class of self-cleaving ribozymes. Both natural sequence variation and the results of mutational analyses support the secondary structure model, including a proposed pseudoknot formed by nucleotides in the loop of P1 and the junction between P2 and P3. This architecture, in addition to the array of 10 highly conserved nucleotides, is distinct from that of all other known self-cleaving ribozymes.

Kinetic analyses of a bimolecular pistol ribozyme construct based on a representative from the bacterium *L. sphaericus* reveal that pistol ribozymes use multiple catalytic strategies to promote an internal phosphoester transfer reaction with a measured rate constant that exceeds the maximum possible for enzymes that only position the RNA linkage for in-line nucleophilic attack (α) and that fully deprotonate the 2′-oxygen nucleophile (γ). If the rate constant enhancements generated by increasing pH (Fig. 3D) and increasing Mg^2+^ concentrations (Fig. 3E) are independent, then the maximum rate constant for the construct tested will be >100 min^−1^, which greatly exceeds the αγ speed limit (Emilsson et al. 2003).

Evidence from the use of a phosphorothioate substrate analog suggests that the ribozyme interacts with one of the two nonbridging phosphate oxygen atoms at the cleavage site, but not via inner-sphere coordination with a divalent metal ion at the active site. Indeed, the results of our metal ion-dependent cleavage assay indicate that pistol ribozymes do not require inner-sphere coordination of divalent metal ions anywhere in the structure, including at the active site, to promote at least modest RNA cleavage activity. However, this pistol ribozyme representative appears to form a contact with one of the two nonbridging oxygen atoms in a way that is strongly disrupted by the presence of sulfur at this critical position.

The simplest explanation for the results of our kinetic and metal ion assays is that pistol ribozymes accelerate RNA cleavage beyond the αγ speed limit by neutralizing the negative charge on one of the two nonbridging phosphate oxygen atoms via interaction with a functional group within the ribozyme RNA, or perhaps by using a water ligand of a hydrated divalent metal ion. Clarification of the precise catalytic strategies used by this ribozyme class will be greatly aided by atomic-resolution structural analyses.

## MATERIALS AND METHODS

### Bioinformatics

The sequence and structural consensus model for pistol ribozymes was established as previously described (Weinberg et al. 2015). This model was updated to include additional microbial and environmental sequences present in RefSeq version 63 and bacteriophage sequences in RefSeq version 64 (Pruitt et al. 2012). Determination (with nucleotide frequencies and covariation) and depiction of the consensus model was achieved by semi-automation using the R2R computer algorithm (Weinberg and Breaker 2011).

### Sample preparation

All substrate RNAs, including those containing deoxyguanosine and phosphorothioate modifications were purchased from Sigma-Aldrich. The phosphorothioate-modified RNA was analyzed by mass spectrometry to verify the presence of the sulfur modification (Fig. 4E). Enzyme RNAs were prepared by in vitro transcription by using T7 RNA polymerase and purified as previously described (Baker et al. 2012) with some exceptions. Double-strand DNA templates for representatives from *Lysinibacillus sphaericus C3-41* (NC_010382.1/2010928-2011021), *Alistipes putredinis* DSM 17216 (NZ_ABFK02000017.1/466361-466281), *Paenibacillus polymyxa ATCC 842 (*NZ_GL905390.1/3135931-3135862), and *Erysipelotrichaceae bacterium 2_2_44A (*NZ_JH126431.1/674694-674757) were generated from overlapping synthetic single-strand DNAs (Sigma-Aldrich) containing a 5′-terminal T7 promoter using SuperScript II reverse transcriptase (Life Technologies). Substrate RNAs were 5′ ^32^P-labeled with [γ-^32^P]ATP (PerkinElmer) and T4 polynucleotide kinase (New England BioLabs) according to the manufacturer's instructions. After transcription or labeling, RNAs were purified by denaturing (8 M urea) 8% (enzyme) or 20% (substrate) PAGE (National Diagnostics).

### Bimolecular cleavage assays

Bimolecular cleavage reactions containing ∼1 nM substrate and 100 nM enzyme were incubated at 23°C in a standard reaction buffer (30 mM HEPES [pH 7.5 at 25°C], 100 mM KCl and 20 mM MgCl_2_), or under different conditions when noted. Reactions were initiated by addition of enzyme RNA and halted at specified times by the addition of three reaction volumes of stop solution (90% formamide, 50 mM EDTA, 0.05% xylene cyanol and 0.05% bromophenol blue). Reaction products were separated by using denaturing (8 M urea) 20% PAGE (National Diagnostics). Gels were imaged and RNA products quantified by using a phosphorimager (Storm Molecular Imager, GE Healthcare Life Sciences). Reactions were performed similarly with mutated RNA substrates, with RNA analogs including the 2′-deoxyguanosine and phosphorothioate substrates, and with mono- and divalent metal ions. For cleavage assays examining the effects of all monovalent metal ions, 100 mM KCl was omitted. All cations tested were chloride salts.

### Mass spectrometry of cleavage products

The *A. putredinis* bimolecular construct was incubated in a 50 μL reaction containing 4 μM enzyme and 3 μM substrate RNAs in 30 mM HEPES (pH 7.5 at 24°C), 100 mM KCl, 20 mM MgCl_2_. The reaction was incubated at 23°C for 30 min and then flash frozen. The sample was subjected to LC-MS for monoisotopic (exact mass) determination (Novatia).

### Rate constant (*k*_obs_) measurements

Cleavage reactions for the Mg^2+^-dependent activity profile were performed at 23°C with 30 mM MES buffer (pH 5.5 at 23°C) and MgCl_2_ concentrations of 0.025 mM to 50 mM. Reactions were initiated by the addition of MgCl_2_. Aliquots of the reaction were removed at various time points and stopped by resuspension in three volumes of stop solution (90% formamide, 50 mM EDTA, 0.05% xylene cyanol, and 0.05 % bromophenol blue). Reactions for the pH-dependent activity profile were performed at 23°C in the appropriate buffer (either 30 mM MES, 30 mM HEPES, or 30 mM Tris–HCl) at a pH range of 5.25–9.0 with 25 μM MgCl_2._ Reactions were initiated and terminated as described above. Cleavage amounts were quantified using ImageQuant software (Molecular Dynamics). Apparent first-order rate constants were determined by linear curve fitting with Excel (Microsoft) using the equation

$$\text{ln}(\;f_{c}/\;f_{\infty })=-k_{\mathrm{obs}}(t)-\;f_{0},$$

where *f*_*c*_ is the fraction cleaved at a specific time point, *f*_∞_ is the maximum possible fraction cleaved, *t* is time, *f*_0_ is the fraction at *t* = 0, and *k*_obs_ is the apparent first-order rate constant.
